# Supplementary material for: MK3 Gene Upregulates Granulosa Cell Apoptosis Through the TNF/P38 MAPK Pathway in Chicken
Source: Cells. 2025 Oct 20;14(20):1630. doi: 10.3390/cells14201630 (PMC12562530; doi:10.3390/cells14201630)
Supplement: Supplementary file 1 [file cells-14-01630-s001.zip › Supplementary table S1.pdf]

Supplementary table S1

Primers for amplifying the core coding regions of *MK3* and *WT1*

Name	primer sequence （5'→3'）	Product size (bp)
PGL-*MK3*-K1	F:cggGGTACCCCATCCCCGTTAGTCCCTTTG	2192
	R:ccgCTCGAGCAGCACTTTGCCGTTGATTC	
PGL-*MK3*-K2	F:cggGGTACCCTTCTCCCGTGGAACAGCGA	1799
	R:ccgCTCGAGCAGCACTTTGCCGTTGATTC	
PGL-*MK3*-K3	F:cggGGTACCTTGTGCCCAGACAACCTATCC	1496
	R:ccgCTCGAGCAGCACTTTGCCGTTGATTC	
PGL-*MK3*-K4	F:cggGGTACCTTTTTCGTCTTCCATCAGCCG	1058
	R: ccgCTCGAGCAGCACTTTGCCGTTGATTC	
PGL-*MK3*-K5	F:cggGGTACCCAGGTAACGTCCTCCGGC	698
	R:ccgCTCGAGCAGCACTTTGCCGTTGATTC	
PGL-*MK3*-K6	F:cggGGTACCGGTCTGACCCCCGTTTGT	308
	R:ccgCTCGAGCAGCACTTTGCCGTTGATTC	
pcDNA3.1-*MK3*	F:cggGGTACCCAGAAGGTCTGCGAGCAG	1140
	R:ccgCTCGAGGTTTATTCACTCGTTTGAAATGC	
pcDNA3.1-*WT1*	F:cggGGTACCATGGAACGCGATGGAGGAG	1130
	ccgCTCGATCATTGGTTATTGCACCCTGG	
