# Supplementary material for: MK3 Gene Upregulates Granulosa Cell Apoptosis Through the TNF/P38 MAPK Pathway in Chicken
Source: Cells. 2025 Oct 20;14(20):1630. doi: 10.3390/cells14201630 (PMC12562530; doi:10.3390/cells14201630)
Supplement: Supplementary file 1 [file cells-14-01630-s001.zip › Supplementary tableS2.pdf]

Supplementary tableS2

The sequences of the siRNAs

Gene Name	forward chain	reverse chain
NC-siRNA	UUCUCCGAACGUGUCACGUTT	ACGUGACACGUUCGGAGAATT
MK3- siRNA - 239	GCCCACGUCAAGCUAGAGATT	UCUCUAGCUUGACGUGGGCTT
MK3- siRNA - 398	GCUCGUCUGGAAGUAGAAUTT	AUUCUACUUCCAGACGAGCTT
MK3- siRNA - 1090	GCCACCAACGCCUCUUCAUTT	AUGAAGAGGCGUUGGUGGCTT
MK3- siRNA - 1192	GGACUACGAUCAGGUGAAATT	UUUCACCUGAUCGUAGUCCTT
