# Supplementary material for: MK3 Gene Upregulates Granulosa Cell Apoptosis Through the TNF/P38 MAPK Pathway in Chicken
Source: Cells. 2025 Oct 20;14(20):1630. doi: 10.3390/cells14201630 (PMC12562530; doi:10.3390/cells14201630)
Supplement: Supplementary file 1 [file cells-14-01630-s001.zip › Supplementary tableS3.pdf]

Supplementary tableS3

Primer sequences used for qPCR

Gene Name	primer sequence（5'→3'）	Product size (bp)	GeneBank accession number
FAS	F：GTTGACCTGACCCACC<br>R：TAGGCTCCTCCCATTC	194	XM_015288355.4
BCL2	F：GCTACCAGAGGGACTTCGC<br>R：GGTCATCCAGGTGGCAAT	215	NM_205339.3
Caspase-3	F：GTGGACCAGATGAAAC<br>R：TGAATAAACCAGGAGC	121	NM_204725.2
C-myc	F：AAGAGGCTAAAGTTGGACAG<br>R：ACGCAGGGCAAAGAAA	168	NM_001030952.2
TNF-R1	F：CCAAAGAGCAGCAAAG<br>R：TGAGTTCAGCCAGTTCC	129	NM_001030779.2
TRAF2	F：AAGGTTGTCGGCTGTG<br>R：GCTCTGTGATGAGGGAAT	162	XM_025141427.3
ASK1	F：TTATGAAGGGACTAACAG<br>R：GTAGGACAGCAGCAAG	275	XM_015284184.4
P 38	F：GTCTCGCACTACATCGTC<br>R：AAACTCCTGGTCACCTATT	117	XM_015296032.4
MK3	F：TATCAGAAGAAGCCAAAC<br>R：TCGTAGTCCACCCTCA	223	NM_001321558.2
