# Supplementary material for: MK3 Gene Upregulates Granulosa Cell Apoptosis Through the TNF/P38 MAPK Pathway in Chicken
Source: Cells. 2025 Oct 20;14(20):1630. doi: 10.3390/cells14201630 (PMC12562530; doi:10.3390/cells14201630)
Supplement: Supplementary file 1 [file cells-14-01630-s001.zip › Supplementary tableS4.pdf]

Supplementary tableS4

The predicted reults of transcription factor binding sites within the core promoter region

Online Sites	transcription factor
JASPAR	JASPAR:Runx1、TFAP2A、WT1、Arnt、Ar、Tbxt、Pax5、、PAX5、NR2F1、CREB1、Cebpa、E2F1、NFIL3、En1、FOXF2、FOXD1
AnimalTFDB 4.0	Sp1、ZNF180、PATZ1、Znf281、Sp3、VEZF1、MAZ、Bcl6、Klf15、Zfp281、WT1 、ASCL1、Glis2、ELF3、TFAP2C、Tfap2a、EGR4、CREB3L4、EBF1、PTF1A、Ar、Myod1、REST、Tcf3
AliBaba 2.1	Sp1、represso、GAL4、YY1、WT1、Krox-20、ETF、RAR-alph、FKBP59、T3R-beta1、COUP、Ttx、ER、Egr-1、Tra-1、AP-2alph、NRF-1、*C-myc*、NF-muE1、CPE_bind
PROMO	MF3、Isl-1、PR-beta、PR-alpha、MyoD、Tal-1、Cdx-1、HOXD8、NF-1、C/EBPalpha、TGGCA-binding protein、AP-3、AP-1
