# Supplementary figures and images for: MK3 Gene Upregulates Granulosa Cell Apoptosis Through the TNF/P38 MAPK Pathway in Chicken
Source: Cells. 2025 Oct 20;14(20):1630. doi: 10.3390/cells14201630 (PMC12562530; doi:10.3390/cells14201630)

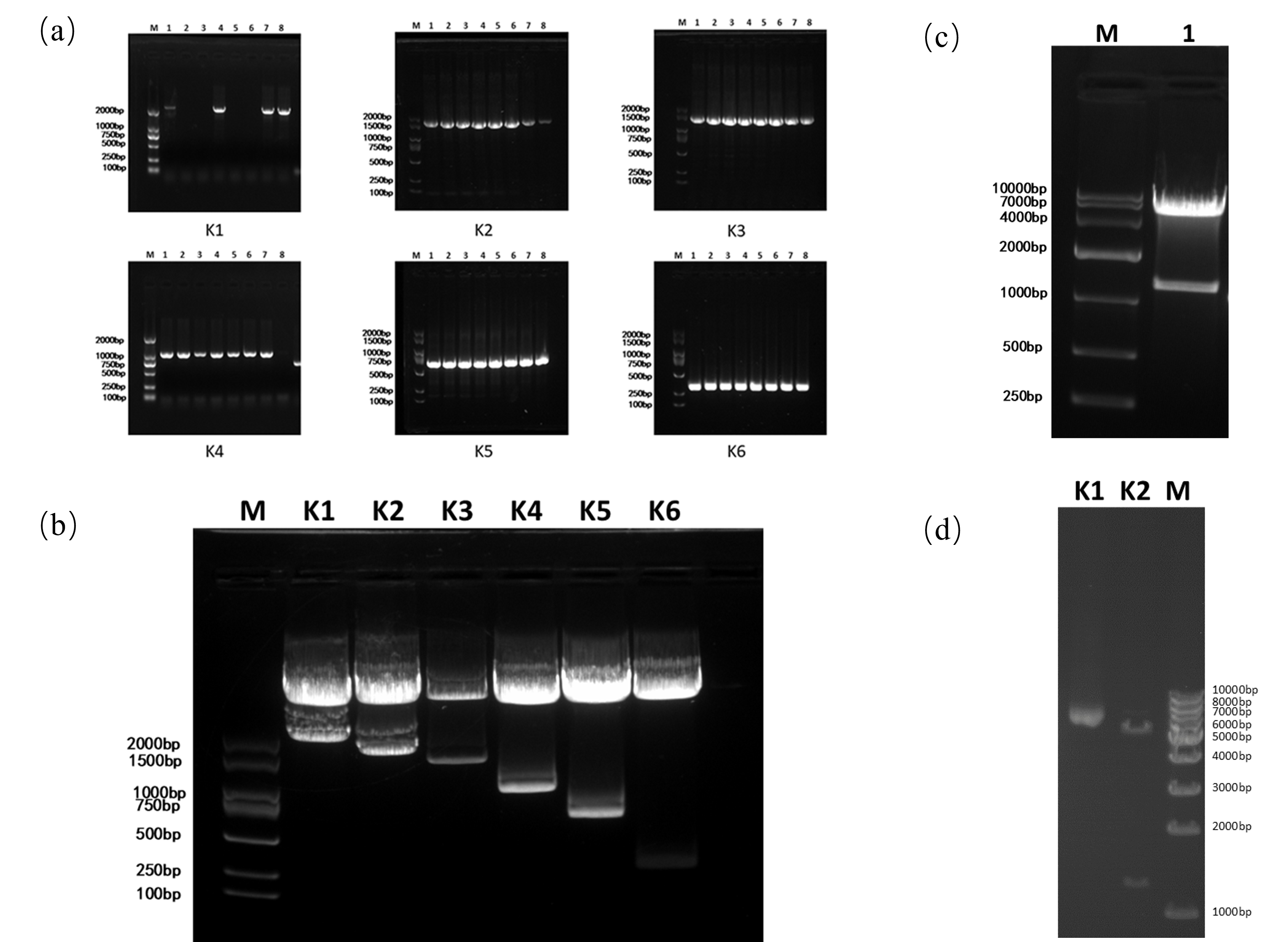

Supplement: Supplementary file 1 [file cells-14-01630-s001.zip › Supplemental Figure S1.tif]
